# Ex Vivo Study of the Vasomotor and Anti-Inflammatory Activity of Class 2 Elastic Venous Compression Devices Combined with Diosmetin-3-O-β-D-Glucuronide

**DOI:** 10.3390/molecules31183142

**Published:** 2026-09-08

**Authors:** Yasmine Belabed, Sylvie Boisnic, Nathaniel Stroumza, Charlotte P. J. Talbot, Yoann Molinard, Frederic Carrois

**Affiliations:** 1 Laboratoire Innotech International, 22 Avenue Aristide Briand, 94110 Arcueil, France; charlotte.talbot@innothera.com (C.P.J.T.); yoann.molinard@innothera.com (Y.M.); frederic.carrois@innothera.com (F.C.); 2 GREDECO (Group of Research and Evaluation in Dermatology and Cosmetology), 69 Rue de la Tour, 75016 Paris, France; sylvie.boisnic@gredeco.com (S.B.); nstroumza@gmail.com (N.S.)

**Keywords:** chronic venous disease, anti-inflammatory, vasomotor activity, diosmin, diosmetin-3-O-β-D-glucuronide, elastic venous compression, skin explant

## Abstract

Diosmin, a flavonoid, is widely used to alleviate the symptoms of chronic venous disease (CVD), while compression stockings remain a key component of its conservative management. This study used an ex vivo model of human skin explants to evaluate the vasomotor and anti-inflammatory effects of a class II elastic venous compression model (EVC2), applied alone or in combination with diosmetin-3-O-β-D-glucuronide (DT), the major circulating metabolite of diosmin. The explants were exposed to substance P to induce neurogenic inflammation, after which they were treated with EVC2, DT at a concentration of 2700 pg/mL, or both. Compared with substance P alone, all treatments led to a significant reduction in capillary dilation and IL-8 secretion, as well as a change in endothelial CD34 expression. While the combination improved all parameters, only significant additional effects were observed for reducing low CD34 expression versus DT alone (77% for score 0, no CD34 expression, *p* = 0.043; 62.1% for score 1, low CD34 expression, *p* = 0.013), and for decreasing IL-8 versus EVC2 alone (41.2%, *p* = 0.033). These findings suggest vasomotor and anti-inflammatory effects of DT and mechanical compression, and highlight their combined effects on inflammatory parameters in CVD.

## 1. Introduction

Chronic venous disease (CVD) is a progressive disorder of the venous system in the lower limbs, characterized by venous hypertension and a wide range of symptoms, including leg heaviness, pain, swelling, edema, and skin changes such as pigmentation or lipodermatosclerosis, and, in advanced stages, ulcerations. These symptoms together can substantially impair patients’ quality of life [1].

Its global prevalence is estimated to be between 10% and 30%, although it occurs most frequently in Western countries [1,2], with a rising trend linked to obesity and an aging population [3].

Although its exact etiology is not fully understood, structural and functional alterations of the venous wall and valves are known to promote venous reflux and/or obstruction. This leads to sustained venous hypertension [4]. These hemodynamic disturbances trigger a series of molecular and cellular processes that contribute to the progression of CVD.

Several mechanisms have been associated with this process, including the activation of inflammatory pathways and matrix remodeling enzymes [3,5]. Matrix metalloproteinases (MMPs) play a central role in degrading extracellular matrix components such as collagen and elastin. This alters venous wall integrity and capillary architecture. Meanwhile, pro-inflammatory cytokines sustain vascular inflammation by maintaining endothelial activation and leukocyte recruitment within the microcirculation [3]. In this context, inflammation and hypoxia are closely interconnected. Venous hypertension and microvascular dysfunction impair oxygen diffusion, leading to local hypoxia, which in turn activates hypoxia-inducible factors (HIFs) and promotes the expression of pro-inflammatory mediators, further amplifying inflammation. Conversely, inflammatory processes exacerbate hypoxia via endothelial damage and capillary rarefaction, creating a self-perpetuating cycle that contributes to oxidative stress, tissue injury, and disease progression [3,6,7].

The treatment of CVD primarily relies on conservative measures such as compression therapy and lifestyle modifications. Surgical or endovenous interventions are reserved for advanced cases [3]. Elastic venous compression is the cornerstone of conservative treatment for CVD and works by increasing interstitial pressure, which reduces the caliber of both superficial and deep veins [6,8]. This mechanical effect decreases venous pressure and edema while promoting the contractile activity in the calf muscles, ultimately improving venous return. Compression stockings are easy to use and are often the first conservative therapy implemented, achieving significant clinical improvements in most patients without substantial discomfort [6]. Additionally, venoactive drugs are commonly used to alleviate symptoms and improve microcirculation. Among these drugs, diosmin, a naturally occurring flavonoid phlebotonic compound, plays a central role [3,4].

Diosmin has venotonic and vasoprotective properties and is indicated to treat symptoms associated with venolymphatic insufficiency, such as heavy legs, pain and nocturnal restlessness. It is also used for the supportive treatment of capillary fragility and the symptoms of hemorrhoidal crisis [9]. The beneficial effects of diosmin are thought to involve anti-inflammatory and antioxidant mechanisms, including inhibiting leukocyte adhesion, suppressing cytokine release, and scavenging of reactive oxygen species [4]. However, its precise molecular mechanisms of action remain incompletely elucidated [3].

After ingestion, diosmin undergoes rapid intestinal hydrolysis, converting it to its aglycone form, diosmetin, which is then absorbed and metabolized into glucuronide conjugates. Of these metabolites, diosmetin-3-O-β-D-glucuronide (DT) is the primary circulating one [10] and is believed to substantially contribute to the pharmacological activity of diosmin in CVD [4]. This metabolite has been shown to have significant anti-inflammatory and antioxidant effects. Maximal responses are observed at 2700 pg/mL, which corresponds to the peak plasma level following the recommended daily dose of 600 mg of diosmin for CVD management [4].

Here, the results of an ex vivo study are presented, in which healthy human skin explants were used to assess the effects of mechanical compression alone or in combination with the major circulating metabolite of diosmin on vasomotor function and inflammatory responses. Explants were maintained under ex vivo survival conditions as an alternative to animal models [11]. To reproduce the physiological conditions of class II elastic venous compression experimentally, a mechanical pressure model was applied to human skin explants after neurogenic inflammation was induced. DT was added to the culture medium at a physiologically relevant concentration, allowing it to diffuse into the dermis under conditions comparable to those observed in vivo after the systemic administration of diosmin in humans [4]. A neurogenic inflammation model was used to mimic stress-induced inflammatory conditions. This model was induced by substance P (SP), the main neuropeptide involved in cutaneous inflammatory responses, which activates neurokinin-1 (NK1) receptors, triggering mast cell degranulation and histamine release. It also induces vascular changes and the secretion of pro-inflammatory cytokines [12,13].

Although the individual effects of diosmin metabolites have been previously described, the physiological responses and pharmacodynamic features within human skin tissue subjected to a class II elastic venous compression (EVC2) model combined with DT exposure has never been explored before in ex vivo conditions. This approach was designed to explore whether mechanical compression and DT may exert complementary effects on vascular and inflammatory parameters, in a setting relevant to the multimodal management of chronic venous disease. As such, the study provides exploratory mechanistic insights while further studies are needed to better assess its translational relevance.

## 2. Results

### 2.1. Assessment of Capillary Dilation Modulation

#### 2.1.1. Quantification of Dilated Capillaries

**Figure 1:**
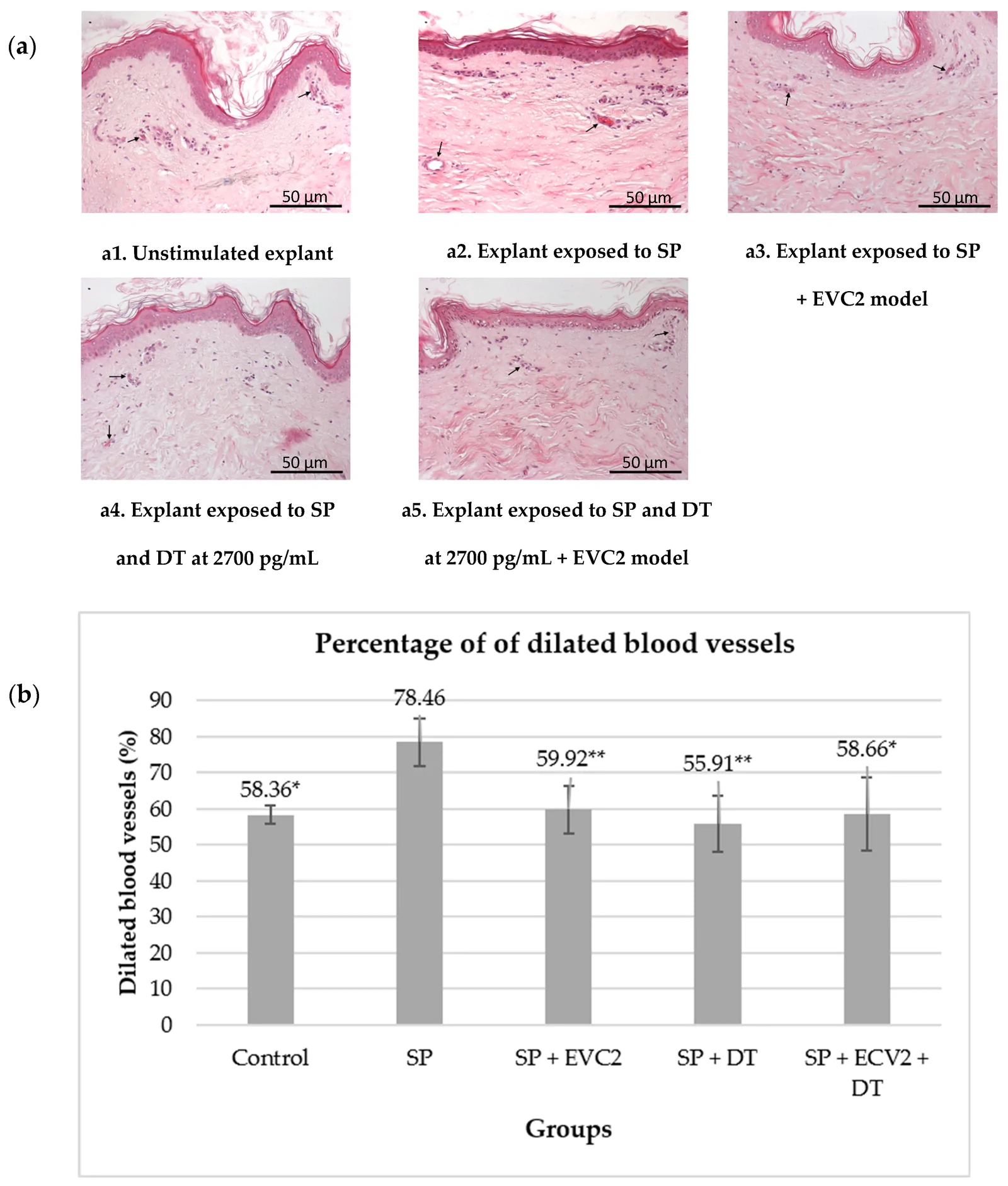
Histological analysis and quantification of capillary dilation in human skin explants. (**a**) Histological visualization of blood capillaries on hematoxylin–eosin–stained sections (×200) of untreated skin (**a1**), skin exposed to SP alone (**a2**), SP plus an EVC2 model (**a3**), SP plus DT at 2700 pg/mL (**a4**), or SP plus EVC2 combined with DT (**a5**), with arrows pointing to blood vessels and the percentage of dilated blood vessels under each condition. Scale bar = 50 μm (**b**). Capillary dilation was defined as a lumen area >40 µm^2^. Histological analyses were performed on two serial sections per donor and per condition. Data are expressed as mean values from five paired biological replicates (*n* = 5 donors), and error bars represent the standard deviation (SD). Symbols indicate statistically significant differences compared with SP (* *p* ≤ 0.05; ** *p* ≤ 0.01). DT: diosmetin-3-O-β-D-glucuronide; EVC2: class 2 elastic venous compression devices; SP: substance P.

Histological analysis revealed that SP exposure resulted in a marked increase in capillary dilation compared to control skin. This increase was subsequently reduced by treatment with EVC2, DT, or a combination of both, relative to SP alone (Figure 1a). Arrows highlight the location of representative vessels, illustrating constricted capillaries in treated conditions versus dilated vessels in the SP condition, which is characterized by luminal enlargement with red blood cell accumulation and a more rounded morphology. A significant increase in the percentage of dilated capillaries was observed following SP-induced neurogenic inflammation compared with control skin (34.4%, *p* = 0.005). Compared to SP alone, EVC2, DT, or their combination significantly reduced the percentage of dilated capillaries by 23.6% (*p* = 0.017), 28.7% (*p* = 0.021), and 25.2% (*p* = 0.003), respectively (Figure 1b). The improvement observed with the combined treatment was not significantly different from that obtained with either treatment alone.

#### 2.1.2. Analysis of the Mean Surface Area of Dilated Blood Capillaries

**Figure 2:**
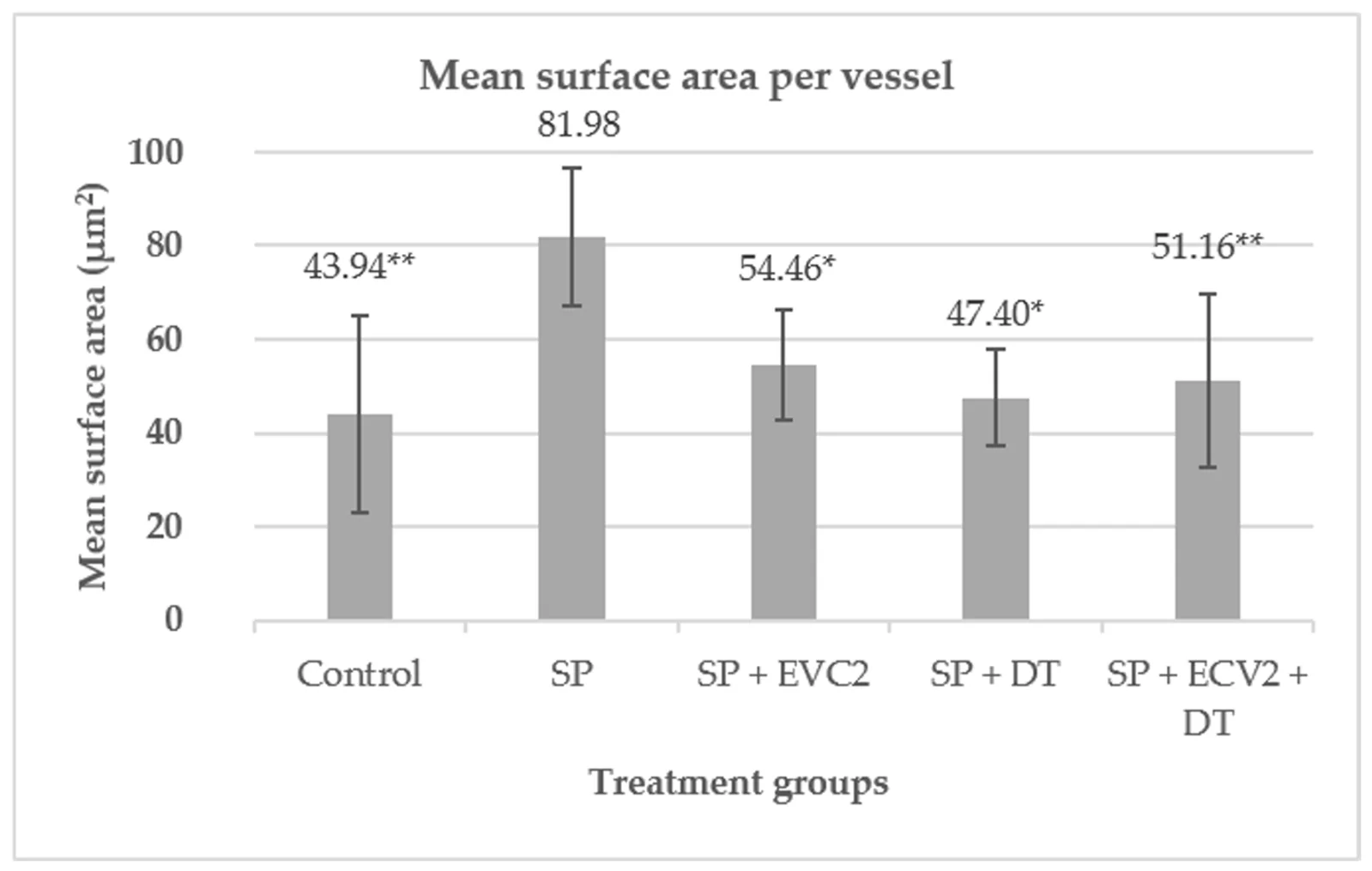
Mean surface area of dilated blood capillaries under different experimental conditions (μm^2^). Data are expressed as mean values from five paired biological replicates (*n* = 5 donors), and error bars represent the standard deviation (SD). Capillary dilation was defined as a lumen area >40 µm^2^. Histological analyses were performed on two serial sections per donor and per condition. Symbols indicate statistically significant differences compared with SP (* *p* ≤ 0.01; ** *p* ≤ 0.001). DT: diosmetin-3-O-β-D-glucuronide; EVC2: class 2 elastic venous compression devices; SP: substance P.

Consistent with the previous findings, histological analysis showed that SP exposure significantly increased the mean surface area of dilated capillaries compared to control skin (86.6%, *p* = 0.001). Treatment with EVC2, DT, or a combination of both resulted in a significant reduction in the mean surface area of dilated capillaries versus SP exposure alone (33.6%, *p* = 0.005; 42.2%, *p* = 0.006; and 37.6%, *p* = 0.001, respectively) (Figure 2). The improvement observed with the combined treatment was not significantly different from the improvements obtained with either treatment alone.

### 2.2. Immunohistochemical Evaluation of CD34 Expression on Endothelial Cells

CD34 expression was evaluated using a semi-quantitative histopathological scoring system (a score of 0 indicates a lack of CD34 labeling on endothelial cells, whereas a score of 3 indicates strong CD34 labeling on endothelial cells) based on the intensity of endothelial immunohistochemical staining, as described in Section 4.

**Figure 3:**
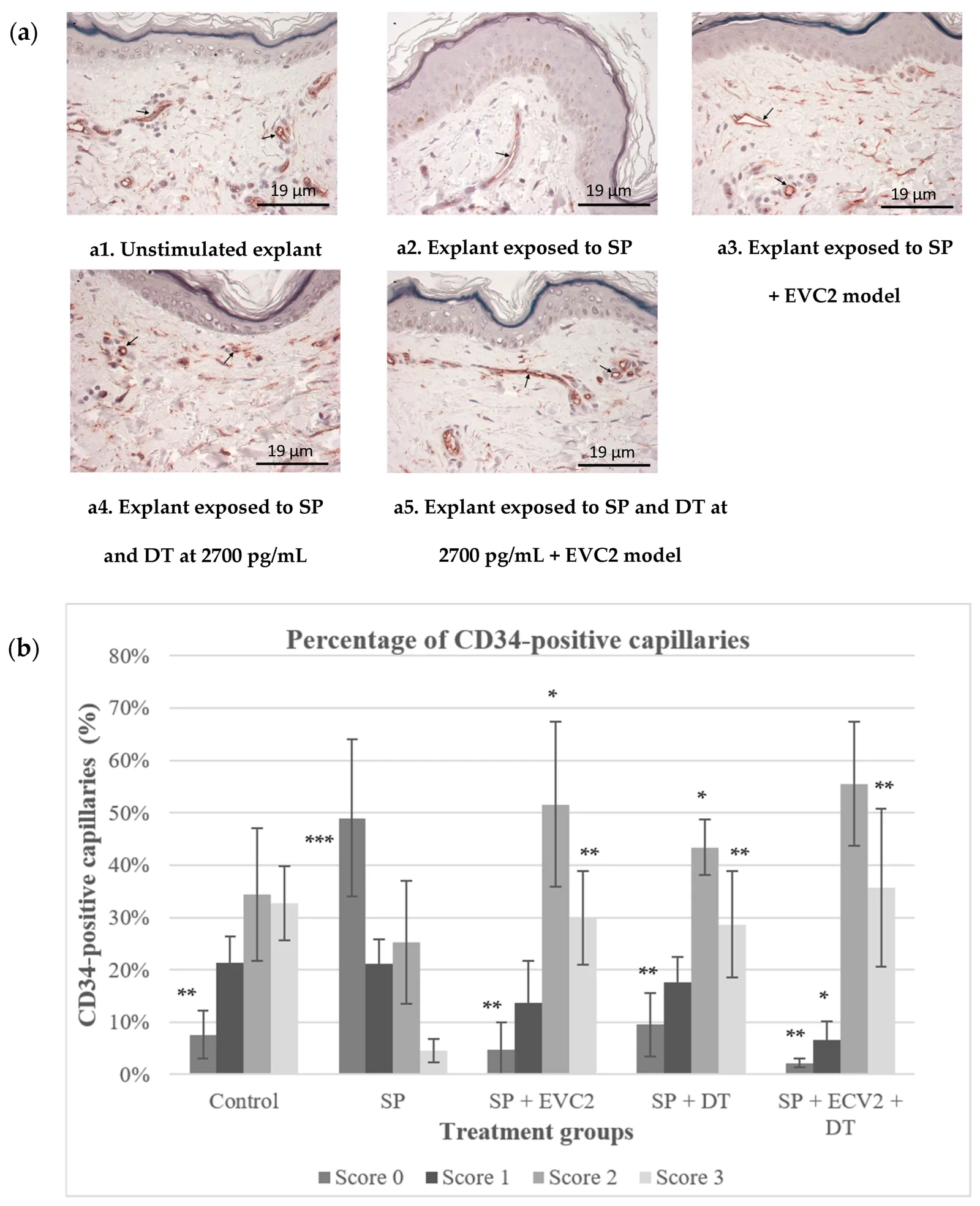
Immunohistochemical assessment of CD34 expression in capillaries. (**a**) Immunohistochemical visualization of vessel labeling with CD34 (×400) of untreated skin fragment (**a1**), fragments exposed to substance P (SP) only (**a2**), to SP and ECV2 (**a3**) or DT at 2700 pg/mL (**a4**) or to SP and ECV2 with DT at 2700 pg/mL (**a5**), with arrows indicating CD34-positive vessels, and percentage of CD34-positive capillaries. Scale bar = 19 μm (**b**). The percentage of CD34-positive capillaries is presented for each experimental condition and distributed according to a semi-quantitative scoring system ranging from 0 to 3, where score 0 indicates the absence of expression, score 1 indicates a low expression, score 2 indicates a moderate expression, and score 3 indicates a strong expression. Histological and immunohistochemical analyses were performed on two serial sections per donor and per condition. Data are expressed as mean values from five paired biological replicates (*n* = 5 donors), and error bars represent the standard deviation (SD). Symbols indicate statistically significant differences compared with SP (* *p* < 0.05; ** *p* < 0.01 and *** *p* ≤ 0.001). DT: diosmetin-3-O-β-D-glucuronide; EVC2: class 2 elastic venous compression devices; SP: substance P.

Following SP exposure, vasodilation (Figure 3a) occurred, which was associated with a redistribution of CD34 expression. This was characterized by a decreased proportion of capillaries with strong CD34 expression (score 3) and an increased proportion in those with no CD34 expression (score 0), compared with control skin (*p* = 0.0006 for score 3; *p* = 0.006 for score 0) (Figure 3b).

Compared to SP alone, treatment with either EVC2 or DT significantly increased the proportion of capillaries with strong CD34 score 3 expression by 6.4-fold increase (*p* = 0.003) and by 6.2-fold increase (*p* = 0.005), respectively, while significantly reducing the proportion of capillaries with no CD34 expression (score 0) by 90.2% (*p* = 0.001) and by 80.4% (*p* = 0.003), respectively (Figure 3b).

Consistent with the effects observed with ECV2 and DT applied after SP exposure, the combination of EVC2 and DT also resulted in a significant increase in the proportion of capillaries exhibiting strong CD34 labeling (score 3) compared with SP alone (7.7-fold increase; *p* = 0.007). Additionally, the combined treatment was associated with a significantly greater reduction in capillaries with low CD34 expression compared with DT alone after SP exposure, with decreases of 77% for score 0 (*p* = 0.043) and 62.1% for score 1 (*p* = 0.013) (Figure 3b).

### 2.3. Biochemical Dosage of Pro-Inflammatory Cytokine (IL-8)

**Figure 4:**
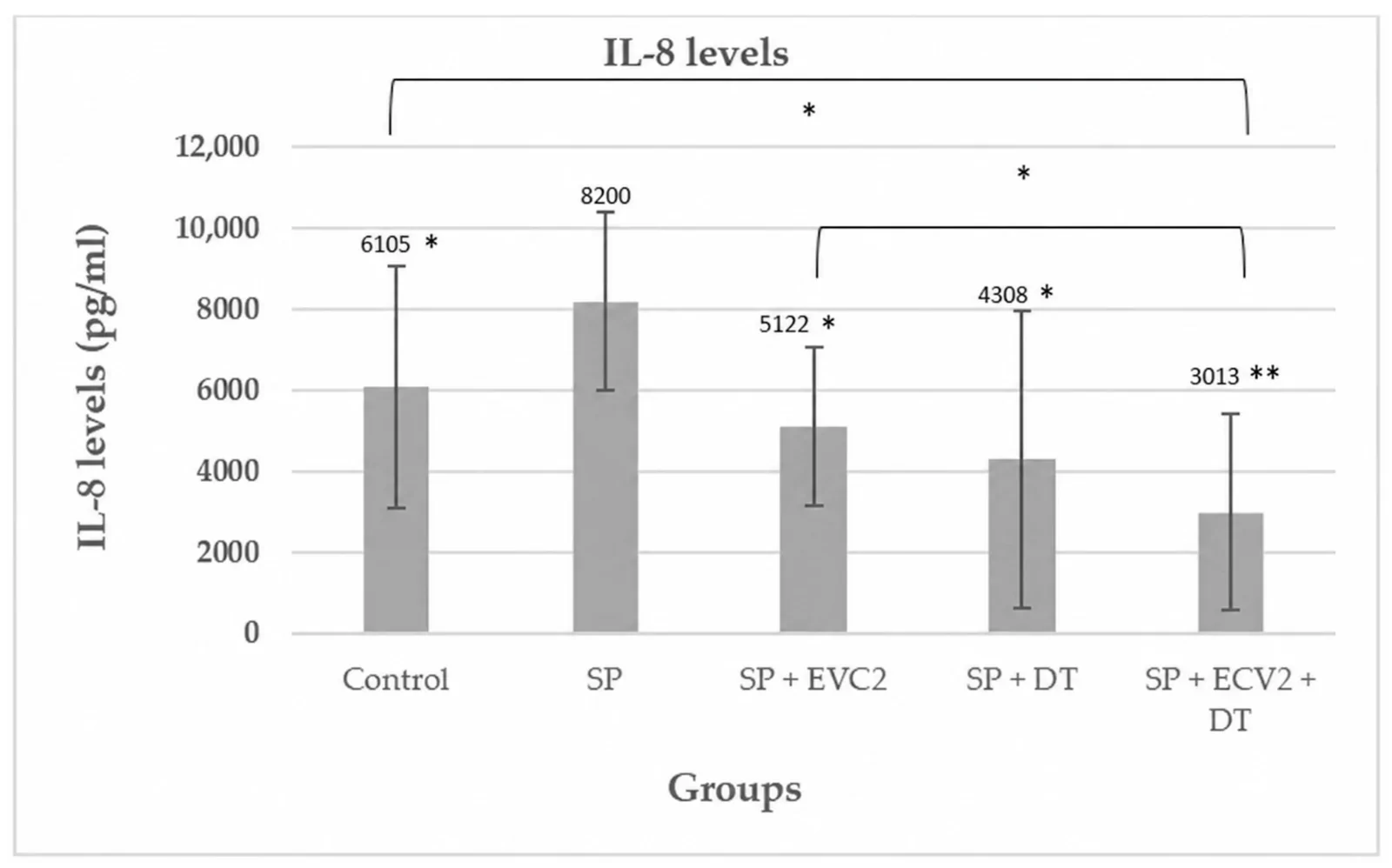
IL-8 levels in the supernatant of skin explants fragments. Pro-inflammatory cytokine IL-8 levels measured in the culture supernatants of human skin explants (*n* = 5 donors) under different experimental conditions: unstimulated control, exposure to SP, and SP exposure and ECV2, DT at 2700 pg/mL, or the combination of EVC2 and DT at 2700 pg/mL. IL-8 measurements were performed in duplicate, and the final value for each donor and condition corresponds to the mean of the duplicate measurements. Data are expressed as mean values, and error bars represent the standard deviation (SD). Symbols without connecting lines indicate statistically significant differences compared with SP, whereas symbols with a connecting line show statistically significant differences between SP + EVC2 and SP + EVC2 + DT, and between control and SP + EVC2 + DT. (* *p* < 0.05; ** *p* < 0.001). DT: diosmetin-3-O-β-D-glucuronide; EVC2: class 2 elastic venous compression devices; SP: substance P.

The neurogenic inflammation model induced by SP resulted in a significant 34.3% increase in IL-8 excretion compared to the control skin (*p* = 0.032). Following SP-induced neurogenic inflammation, treatment with EVC2 or DT significantly reduced IL-8 levels by 37.5% (*p* = 0.011) and 47.5% (*p* = 0.018), respectively. The reduction observed with the combined treatment was significant as well (63.3%, *p* = 0.0003), and it was associated with a significantly greater reduction compared with EVC2 alone (41.2%, *p* = 0.033) (Figure 4).

## 3. Discussion

The aim of this study was to evaluate the vasomotor and anti-inflammatory effects of a pressure model mimicking class II elastic venous compression (EVC2), applied alone or in combination with diosmetin-3-O-β-D-glucuronide (DT). DT is the major circulating metabolite of diosmin and was tested at a concentration of 2700 pg/mL. In this study, human skin explant models derived from five donors and maintained under ex vivo survival conditions were exposed to SP to induce neurogenic inflammatory responses.

CVD is a progressive condition driven by sustained venous hypertension and alterations in venous hemodynamics [6]. These disturbances promote venous dilation, blood stasis, and microcirculatory dysfunction, initiating and maintaining a local inflammatory response. Inflammation is now recognized as a hallmark of CVD [14]. It involves endothelial activation followed by the recruitment and infiltration of immune cells, including leukocytes, monocytes/macrophages, mast cells, and lymphocytes. These immune cells contribute to the release of pro-inflammatory mediators, such as cytokines and chemokines, including, but not limited to, IL-8, IL-6, or TNF-alpha, thereby sustaining endothelial dysfunction, tissue hypoxia, oxidative stress, and extracellular matrix remodeling [6,7]. Together, these mechanisms create a self-perpetuating inflammatory cycle that leads to venous wall damage and disease progression.

Although the pharmacological use of diosmin has long been supported by clinical data demonstrating improvements in venous tone and symptoms [15,16], the precise mechanisms underlying its endothelial and anti-inflammatory effects have remained incompletely understood. The present ex vivo findings provide new mechanistic insights into the pharmacodynamics of diosmin.

Both EVC2 and DT independently exerted a significant vasomotor effect under stress (SP) conditions, as demonstrated by a reduction in capillary dilation and in the mean luminal surface area of dilated capillaries compared to stress alone. The observed vasomotor effect with DT is consistent with previous findings obtained using the same ex vivo model [4]. In that study, diosmetin-3-O-β-D-glucuronide significantly reduced both the proportion of dilated capillaries and their mean luminal cross-sectional area at all tested concentrations (*p* < 0.0001), suggesting a normalization effect on capillary tone and supporting the compound’s intrinsic vasculoprotective and venotonic activity [4]. Interestingly, the combined treatment (EVC2 + DT) also significantly reduced these histological parameters. However, no additional benefit was observed compared to the treatments administered alone, indicating there was no greater effect on capillary dilation outcomes.

The CD34 antigen is a well-established marker of the vascular endothelium and neovascularization [17]. CD34 immunostaining was therefore performed to assess endothelial expression patterns as part of the vascular analysis. Under stress conditions, a pronounced decrease in endothelial CD34 expression was observed, reflected by a reduction in capillaries with strong CD34 expression (score 3) and an increase in those with no expression (score 0). EVC2 and DT both markedly restored CD34 expression (increased score of 3 vs. SP) and reduced the proportion of capillaries with no expression (score 0). Importantly, the combined EVC2 + DT treatment produced a significantly greater reduction in low-expression categories (scores 0 and 1) compared to DT alone. This indicates an enhanced endothelial response under stress conditions.

This restoration is in line with the previously reported effects of diosmin on hypoxia-associated and angiogenic regulatory pathways in humans. Clinical studies have shown that a three-month oral administration of diosmin significantly reduced proangiogenic factors such as vascular endothelial growth factors A and C (VEGF-A and VEGF-C), and fibroblast growth factor 2 (FGF-2) while increasing angiostatin and endostatin levels [3,16]. This suggests a shift toward functional rather than pathological angiogenesis. Additionally, diosmin has been reported to decrease hypoxia-related markers, including HIF and lactate levels, in patients with CVD [3], as well as to preserve endothelial function and glycocalyx integrity, thereby limiting vascular permeability and inflammation in preclinical models [18]. In addition, mechanical compression improves venous hemodynamics by reducing venous diameter, pressure, and edema. This promotes venous return and alleviates venous hypertension, which may secondarily support endothelial recovery under stressful conditions [6]. Taken together, these observations suggest that diosmin and its major circulating metabolite support endothelial recovery and vascular protection, potentially contributing to the normalization of tissue conditions associated with hypoxia. The combined EVC2 plus DT treatment appears to reinforce these effects under inflammatory stress, supporting the relevance of associating mechanical compression with diosmin therapy in the context of CVD.

In the present ex vivo model of substance P–induced inflammation, EVC2 alone significantly reduced IL-8 levels by 37.5% (*p* = 0.011), demonstrating the anti-inflammatory potential of mechanical compression. DT alone also produced a significant decrease of 47.5% in IL-8 secretion (*p* = 0.018). These results are consistent with previously published data obtained using the same experimental model, where DT induced a significant IL-8 reduction at the physiologically relevant concentration of 2700 pg/mL [4], thereby confirming the intrinsic anti-inflammatory potential of DT.

Notably, our results further demonstrated that the combined EVC2 + DT treatment resulted in a significantly greater decrease in IL-8 (63.3%, *p* = 0.0003) compared to EVC2 alone (Student’s *t*-test, *p* = 0.033). This enhanced reduction suggests that mechanical compression and DT may have complementary anti-inflammatory effects in this model. This is consistent with the known intrinsic anti-inflammatory properties of diosmin mediated through its major circulating metabolite DT.

Beyond the present findings, several in vitro and in vivo studies support the anti-inflammatory potential of diosmin and its aglycone, diosmetin. Diosmin has been shown to reduce the production and gene expression of pro-inflammatory mediators, including nitric oxide (NO), prostaglandin E2 (PGE2), interleukins 6 and 12 (IL-6 and IL-12), and tumor necrosis factor α (TNF-α), by inhibiting the nuclear factor-kappa B (NF-κB) and mitogen-activated protein kinases (MAPK) signaling pathways [19,20]. Similarly, both diosmin and diosmetin were reported to reduce the levels of IL-6, interleukin 1β (IL-1β), cyclooxygenase 2 (COX-2), and PGE2 levels in lipopolysaccharide-stimulated human skin fibroblasts, with diosmetin exhibiting a stronger effect [21]. These molecular findings complement the present results and support the hypothesis that diosmin and its major circulating metabolite have direct and indirect anti-inflammatory effects at the endothelial and tissue levels [16,22].

The present study has several limitations. Although this previously established ex vivo model does not fully reproduce the multifactorial and chronic pathophysiology of chronic venous disease, it provides a controlled and reproducible platform for investigating early vascular and inflammatory responses. The use of healthy human skin explants stimulated with substance P enables the evaluation of tissue-specific biological mechanisms under standardized experimental conditions. Accordingly, our findings should be interpreted as observations in an induced inflammatory model, while their relevance to the chronic clinical setting should be confirmed in complementary in vivo studies. In addition, as this study investigates effects on skin tissue, plasma concentrations of the compound may not accurately reflect its exposure at the tissue level. The sample size was small because the ex vivo experiments were conducted on skin explants obtained from a limited number of donors (*n* = 5). While this limitation is inherent to human ex vivo models, it may restrict the generalizability of the findings. Although paired analyses were appropriate in this ex vivo design, the limited sample size reduces statistical power, and supports an exploratory interpretation of the findings. Larger studies that include more donors will be needed to confirm these results. Only a few biological parameters were examined, with a primary focus on vasomotor and inflammatory endpoints. Additional molecular or cellular mechanisms that may be involved in the effects of elastic venous compression and diosmetin were therefore not evaluated and warrant further investigation. In this study, the inflammatory assessment focused on IL-8, which is a relevant marker in the present neurogenic inflammation model. Additional markers of endothelial activation (e.g., ICAM-1, VCAM-1) and inflammation (e.g., NF-κB, COX-2, IL-6, TNF-α, MCP-1) could provide further mechanistic insights and should be explored in future studies. Furthermore, the ex vivo compression model used in this study was designed to investigate the early biological effects of compression under controlled conditions. In this model, the applied pressure was estimated theoretically from standard mmHg to g/cm^2^ conversion equivalences. Although this provided a standardized approach to compression, the actual pressure distribution across the skin fragment was not directly measured. Although it does not fully reproduce the complexity of clinical compression therapy, the findings provide relevant mechanistic insights that should be confirmed in complementary in vivo studies. Finally, the present study did not include control groups treated with DT alone or EVC2 alone in the absence of substance P. Therefore, we cannot exclude potential effects of these interventions on basal vascular or inflammatory parameters. This limitation should be considered in the present study, and future work should address these conditions.

Despite these limitations, the data obtained from the vasomotor, endothelial, and inflammatory analyses in this ex vivo model support a dual mode of action for diosmin and its major circulating metabolite. Diosmetin-3-O-β-D-glucuronide acts at the endothelial interface, as indicated by increased CD34 expression, while simultaneously reducing the release of inflammatory mediators (IL-8). The simultaneous increase in high CD34 endothelial expression and the greater reduction in IL-8 secretion under combined EVC2 + DT treatment suggest complementary pharmacodynamic effects, with concurrent modulation of CD34 expression and reduction of inflammatory marker secretion. However, given the exploratory nature of this ex vivo model, these findings support a favorable modulation of CD34 expression, whose functional significance warrants further investigation in functional models. Furthermore, it should be noted that DT alone produced significant effects in all tested parameters, confirming its full pharmacological activity and reinforcing its role as the major circulating metabolite of diosmin. Combining it with EVC2 further enhanced selected endothelial marker expression and inflammatory outcomes, supporting the relevance of integrating mechanical compression with pharmacological intervention to modulate CD34 expression and control inflammation in CVD.

## 4. Materials and Methods

### 4.1. Culture of Human Skin Fragments

Healthy skin explants were obtained from five female Caucasian donors aged 48 to 60 years. Tissue samples were collected from reduction mammoplasty, brachioplasty, and abdominoplasty procedures. Donor sites were selected to ensure the presence of a sufficient number of vessels for histological analysis. For each donor, one skin explant culture was performed per condition. Because each donor served as their own control in the ex vivo design, donor-to-donor variability was partially controlled. Informed consent was obtained from each donor prior to study initiation, allowing the anonymous donation of skin fragments for research purposes, in compliance with the General Data Protection Regulation (GDPR).

The human skin organ culture model preserves the structural integrity of the skin and is therefore considered to closely reflect in vivo human skin responses [23].

After excision, the skin fragments were placed in culture inserts with the epidermis facing upward, on a polycarbonate membrane with 3 µm-pore positioned in culture wells (Costar, VWR, Fontenay-sous-Bois, France). The inserts were then placed in multiwell plates containing culture medium added exclusively to the lower chamber, allowing diffusion between the two compartments that were separated by the porous membrane.

The culture medium consisted of Dulbecco’s Modified Eagle’s Medium (Gibco BRL, Grand Island, NY, USA), supplemented with antibiotics (penicillin 100 µg/mL, streptomycin 100 µg/mL, amphotericin B 250 µg/mL), hormones (Sigma, Saint-Quentin-Fallavier, France), bovine pituitary extract (Gibco BRL, Grand Island, NY, USA), and fetal calf serum (DAP, Le Bourget, France). The cultures were maintained at 37 °C in a humidified atmosphere containing 5% CO_2_.

### 4.2. Diosmetin 3-O-β-D-Glucuronide

DT (diosmetin 3-glucuroconjugate) was commercially obtained from Syncom (Groningen, The Netherlands; Syncom number 32429; CAS 152503-50-9). According to the supplier’s certificate of analysis, the compound had a molecular formula of C_22_H_24_O_12_, a molecular weight of 476.39, and a purity of 97.3% at 215 nm and 96.7% at 346 nm by HPLC-MS. It was supplied as a yellow solid (batch no. 1785). It was dissolved in dimethyl sulfoxide (DMSO) to create a stock solution of 2 mg/mL.

An intermediate dilution of 1:10 in phosphate-buffered saline solution (PBS) was then prepared to yield a 200 µg/mL solution, minimizing any potential cytotoxic effect of DMSO on skin fragments.

The DT was added to the culture medium at a final concentration of 2700 pg/mL, immediately upon initiation of the explant survival phase, and reapplied after induction of neurogenic inflammation and implementation of the compression model.

This concentration was chosen to approximately reflect a physiologically relevant exposure. It is in the range of the plasma levels of DT reported in humans following oral administration of 600 mg of diosmin, and is consistent with those used in previously described human skin ex vivo models [4].

### 4.3. Substance P-Induced Inflammatory Stress Model

To evaluate the anti-inflammatory effect of the tested product, a neurogenic inflammation model was established using SP (ref. H-1890, Bachem, Bubendorf, Switzerland), the main neuropeptide involved in the initiation of cutaneous inflammatory responses.

This model reproduces the mechanisms of neurogenic inflammation, including vascular alterations (edema and vasodilation) and the release of pro-inflammatory cytokines in human skin explants maintained under survival conditions [12]. It also mimics the local inflammatory processes that contribute to pain in CVD [24].

After a two-hour survival period under standard culture conditions, the skin fragments were exposed to substance P at a final concentration of 10 µM, according to the previously described methodology [12].

Depending on the experimental condition, the skin fragments were immediately exposed after SP stimulation to either class II elastic venous compression (EVC2), DT treatment, or the combination of both.

### 4.4. Mechanical Compression Model

A mechanical pressure model was developed to reproduce EVC2, corresponding to a range of 15–20 mmHg [25]. Based on standard pressure-to-weight equivalences (10 mmHg ≈ 13.6 g/cm^2^; 36 mmHg ≈ 48.9 g/cm^2^), the class II compression range of 15–20 mmHg corresponds to approximately 20.4–27.2 g/cm^2^. Therefore, a total weight of 25 g was applied to each 1 cm^2^ skin fragment. Of note, the applied weight was derived from standard pressure-to-weight conversion equivalences, without experimental measurement or mapping on the skin surface.

Compression was applied after two hours of explant survival, immediately following the induction of neurogenic inflammation by substance P and maintained on the skin during 24 h.

### 4.5. Experimental Conditions

**Figure 5:**
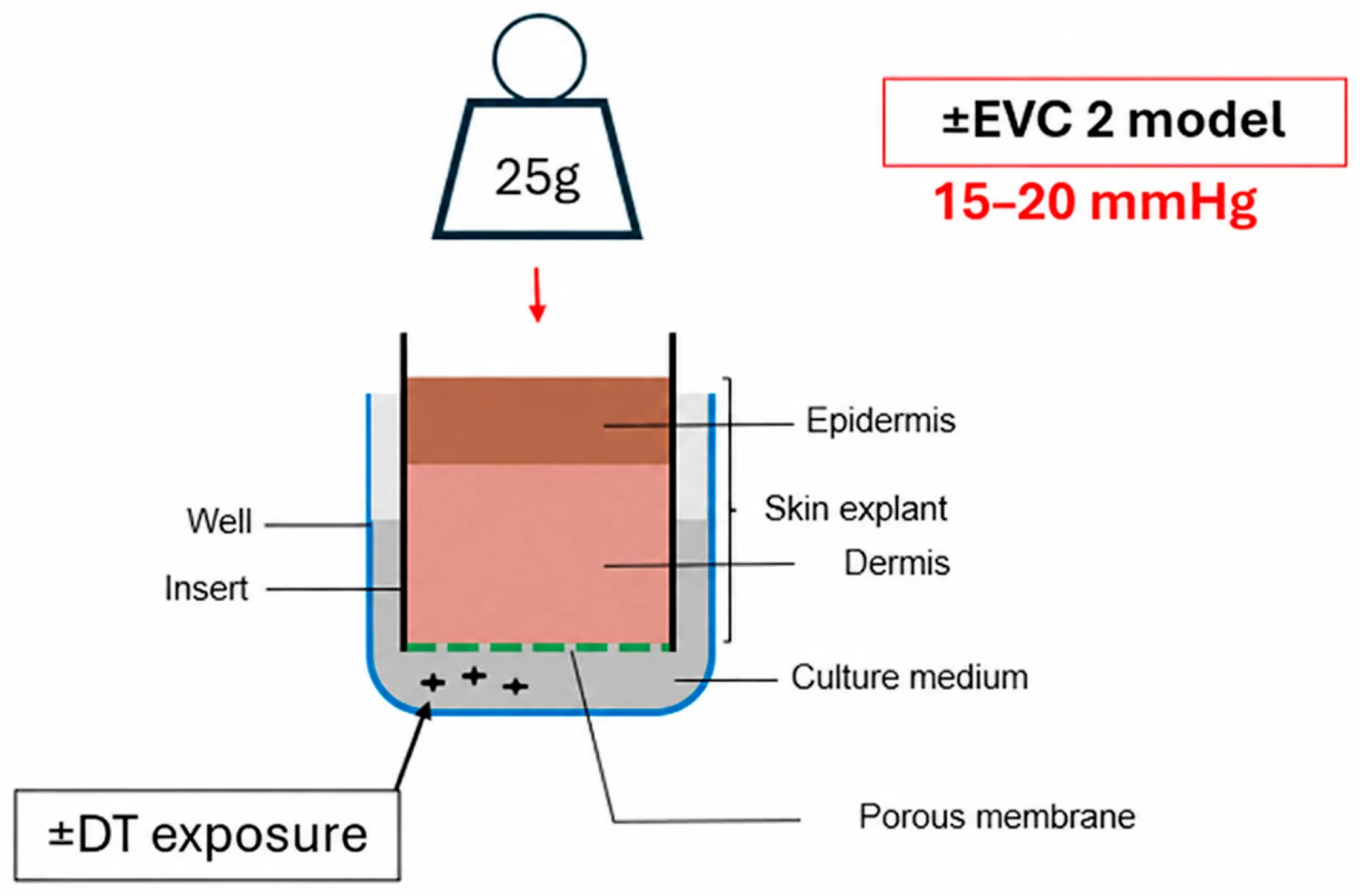
Experimental model: skin explant fragments were placed with the epidermis facing upward on the porous membrane of tissue culture inserts set in wells containing culture medium. The explants were exposed to substance P (SP) and subsequently subjected to a class II elastic venous compression model (EVC2), a treatment with diosmetin-3-O-β-D-glucuronide (DT) at 2700 pg/mL added to the culture medium, or a combination of both treatments.

For each donor, comparative analyses were performed under five experimental conditions (Figure 5):

- Untreated control skin;
- Skin stimulated with substance P;
- Skin stimulated with substance P and exposed to EVC2;
- Skin stimulated with substance P and treated with DT (2700 pg/mL);
- Skin stimulated with substance P, treated with DT (2700 pg/mL), and exposed to EVC2.

Skin fragments were maintained in culture for 24 h.

### 4.6. Evaluated Parameters

Morphological, immunohistochemical, and biochemical parameters were assessed to characterize the effects of SP inflammatory stress, as well as those of mechanical compression and/or DT treatment.

#### 4.6.1. Histological Analysis of the Dermal Capillary Network

At the end of the culture period, the skin fragments were fixed in formalin, embedded in paraffin, sectioned, and stained with hematoxylin–eosin for histological analysis.

Vascular dilation was assessed by counting the number of dilated capillaries across the entire histological section at 400× magnification. This number was expressed as a percentage of the total number of capillaries.

A morphometric computerized analysis of the capillary surface area (µm^2^) was also performed to determine the mean area occupied by capillaries in the dermis.

Additionally, the total number of capillaries was counted to assess potential neovascularization, and the mean number of capillaries per field was calculated.

Quantitative histological analysis was performed on two sections per donor to verify slice uniformity. Approximately 15 microscopic fields and 100 to 150 capillaries were evaluated per sample using an Olympus BX41 microscope (Olympus France, Rungis, France) and CellSens V3 software. The threshold used to define capillary dilation was a lumen area greater than 40 µm^2^. The analysis was performed by Dr. Sylvie Boisnic, MD, anatomopathologist, and was not blinded.

#### 4.6.2. Immunohistochemical Assessment of CD34 Expression

The CD34 antigen is a sensitive marker of vascular endothelium and angiogenesis [15]. The expression of CD34, which is a marker of dermal capillary endothelial cells, was assessed by immunohistochemistry using a class II anti-CD34 antibody (clone QBEnd 10, Dako, Glostrup, Denmark). Immunodetection was performed using a Polink 2 Plus kit (GBI) and visualized with an aminoethyl carbazole (AEC) chromogen.

The intensity of CD34 expression was assessed using a semi-quantitative score (0–3), where score 0 indicates the absence of expression, score 1 indicates a low expression, score 2 indicates a moderate expression, and score 3 indicates a strong expression on endothelial cells. To determine the proportion of CD34-positive vessels, the number of capillaries corresponding to each score was expressed as a percentage of the total number of dermal capillaries.

CD34 immunostaining was evaluated by a single observer (Dr. Boisnic, MD, anatomopathologist) using an Olympus BX41 microscope. Between 80 and 100 CD34-positive capillaries were assessed per sample.

#### 4.6.3. Quantification of Interleukin-8 (IL-8)

Culture supernatants were collected at the end of the incubation period and stored at −32 °C until analysis. Interleukin-8 (IL-8) concentrations were measured using a commercially available enzyme-linked immunosorbent assay (ELISA) kit (Human IL-8/CXCL8 DuoSet ELISA kit, Ref. DY6220-5, Bio-Techne, Dublin, Ireland), according to the manufacturer’s instructions. Results were expressed in pg/mL.

### 4.7. Statistical Analysis

The results were analyzed using data obtained from the five human skin samples. The Shapiro–Wilk test was used to assess the normality of data distributions. Comparisons were performed using a paired Student’s *t*-test when normality was confirmed, or a Wilcoxon signed-rank test in the absence of normality. The significance level (α) was set at 5%. Statistical analysis was performed using GraphPad Prism software (version 10.1.1; GraphPad Software, San Diego, CA, USA).

## 5. Conclusions

Our ex vivo results indicate that diosmetin-3-O-β-D-glucuronide, the major circulating metabolite of diosmin, has significant anti-inflammatory and vasomotor effects at concentrations similar to those achieved with the oral intake of 600 mg of diosmin. Similar vasomotor and anti-inflammatory effects were induced by mechanical compression, consistent with its established role in improving venous hemodynamics. Combining diosmetin-3-O-β-D-glucuronide and class II elastic venous compression showed selective combined effects, particularly reinforcing the anti-inflammatory response. However, these findings were obtained in an acute ex vivo model using human skin explants and should be considered exploratory. Further studies, including additional inflammatory markers and complementary models, will be valuable to better characterize the pharmacological mechanisms underlying the effects of diosmin in chronic venous disease.

## Data Availability

The data presented in this study are available on request from the corresponding author.

## Abbreviations

The following abbreviations are used in this manuscript:

AEC	Aminoethyl carbazole
COX-2	Cyclooxygenase 2
CVD	Chronic venous disease
DMSO	Dimethyl sulfoxide
DT	Diosmetin-3-O-β-D-glucuronide
ELISA	Enzyme-linked immunosorbent assay
EVC2	Class 2 elastic venous compression devices
FGF-2	Fibroblast growth factor 2
GDPR	General Data Protection Regulation
HIF	Hypoxia inducible factor
IL-12	Interleukin 12
IL-1β	Interleukin 1β
IL-6	Interleukin 6
IL-8	Interleukin 8
MAPK	Mitogen-activated protein kinases
MMP	Matrix metalloproteinase
NF-κB	Nuclear factor-kappa B
NK1	Neurokinin-1 receptor
NO	Nitric oxide
PBS	Phosphate-buffered saline solution
PGE2	Prostaglandin E2
SP	Substance P
TNF	Tumor necrosis factor
VEGF-A	Vascular endothelial growth factor A
VEGF-C	Vascular endothelial growth factor C

## References

[1] Davies A.H. (2019). The Seriousness of Chronic Venous Disease: A Review of Real-World Evidence. Adv. Ther..

[2] Feldo M., Wójciak M., Sowa I., Przywara S., Sowa P., Paduch R. (2023). Effect of diosmin and diosmetin on the level of pro-inflammatory factors in the endothelium artificially induced with inflammatory stimuli. Acta Angiol..

[3] Feldo M., Wójciak M., Dresler S., Sowa P., Płachno B.J., Samborski D., Sowa I. (2023). Effect of Diosmin on Selected Parameters of Oxygen Homeostasis. Int. J. Mol. Sci..

[4] Boisnic S., Branchet M.C., Quioc-Salomon B., Doan J., Delva C., Gendron C. (2023). Anti-Inflammatory and Antioxidant Effects of Diosmetin-3-O-β-d-Glucuronide, the Main Metabolite of Diosmin: Evidence from Ex Vivo Human Skin Models. Molecules.

[5] Santoliquido A., Carnuccio C., Santoro L., Di Giorgio A., D’Alessandro A., Ponziani F.R., Angelini F., Izzo M., Nesci A. (2025). Local and Systemic Endothelial Damage in Patients with CEAP C2 Chronic Venous Insufficiency: Role of Mesoglycan. Int. J. Mol. Sci..

[6] Ortega M.A., Fraile-Martínez O., García-Montero C., Álvarez-Mon M.A., Chaowen C., Ruiz-Grande F., Pekarek L., Monserrat J., Asúnsolo A., García-Honduvilla N. (2021). Understanding Chronic Venous Disease: A Critical Overview of Its Pathophysiology and Medical Management. J. Clin. Med..

[7] Fraile-Martinez O., García-Montero C., Gomez-Lahoz A.M., Sainz F., Bujan J., Barrena-Blázquez S., López-González L., Díaz-Pedrero R., Álvarez-Mon M., García-Honduvilla N. (2025). Evidence of Inflammatory Network Disruption in Chronic Venous Disease: An Analysis of Circulating Cytokines and Chemokines. Biomedicines.

[8] Rabe E., Partsch H., Morrison N., Meissner M.H., Mosti G., Lattimer C.R., Carpentier P.H., Gaillard S., Jünger M., Urbanek T. (2020). Risks and contraindications of medical compression treatment—A critical reappraisal. An international consensus statement. Phlebology.

[9] Savineau J.P., Marthan R. (1994). Diosmin-induced increase in sensitivity to Ca^2+^ of the smooth muscle contractile apparatus in the rat isolated femoral vein. Br. J. Pharmacol..

[10] Silvestro L., Tarcomnicu I., Dulea C., Attili N.R.B.N., Ciuca V., Peru D., Savu S.R. (2013). Confirmation of diosmetin 3-O-glucuronide as major metabolite of diosmin in humans, using micro-liquid-chromatography–mass spectrometry and ion mobility mass spectrometry. Anal. Bioanal. Chem..

[11] Boisnic S., Branchet-Gumila M.C., Benslama L., Charpentier Y.L., Arnaud-Battandier J. (1997). Long term culture of normal skin to test the efficacy of a hydroxy acid-containing cream. Eur. J. Dermatol..

[12] Branchet-Gumila M.C., Boisnic S., Charpentier Y.L., Nonotte I., Montastier C., Breton L. (1999). Neurogenic Modifications Induced by Substance P in an Organ Culture of Human Skin. Ski. Pharmacol. Physiol..

[13] Mashaghi A., Marmalidou A., Tehrani M., Grace P.M., Pothoulakis C., Dana R. (2016). Neuropeptide Substance P and the Immune Response. Cell Mol. Life Sci..

[14] Petrascu F.M., Matei S.C., Margan M.M., Ungureanu A.M., Olteanu G.E., Murariu M.S., Olariu S., Marian C. (2024). The Impact of Inflammatory Markers and Obesity in Chronic Venous Disease. Biomedicines.

[15] Feldo M., Woźniak M., Wójciak-Kosior M., Sowa I., Kot-Waśik A., Aszyk J., Bogucki J., Zubilewicz T., Bogucka-Kocka A. (2018). Influence of Diosmin Treatment on the Level of Oxidative Stress Markers in Patients with Chronic Venous Insufficiency. Oxidative Med. Cell. Longev..

[16] Feldo M., Wójciak-Kosior M., Sowa I., Kocki J., Bogucki J., Zubilewicz T., Kęsik J., Bogucka-Kocka A. (2019). Effect of Diosmin Administration in Patients with Chronic Venous Disorders on Selected Factors Affecting Angiogenesis. Molecules.

[17] Yoshida H., Fujita S., Nishida M., Iizuka T. (1999). Angiogenesis in the human temporomandibular joint studied by immunohistochemistry for CD34 antigen. J. Oral Pathol. Med..

[18] Mitra R., Nersesyan A., Pentland K., Melin M.M., Levy R.M., Ebong E.E. (2022). Diosmin and its glycocalyx restorative and anti-inflammatory effects on injured blood vessels. FASEB J..

[19] Berkoz M. (2019). Diosmin suppresses the proinflammatory mediators in lipopolysaccharide-induced RAW264.7 macrophages via NF-κB and MAPKs signal pathways. Gen. Physiol. Biophys..

[20] Imam F., Al-Harbi N.O., Al-Harbi M.M., Ansari M.A., Zoheir K.M.A., Iqbal M., Anwer K., Al Hoshani A.R., Attia S.M., Ahmad S.F. (2015). Diosmin downregulates the expression of T cell receptors, pro-inflammatory cytokines and NF-κB activation against LPS-induced acute lung injury in mice. Pharmacol. Res..

[21] Feldo M., Wójciak M., Ziemlewska A., Dresler S., Sowa I. (2022). Modulatory Effect of Diosmin and Diosmetin on Metalloproteinase Activity and Inflammatory Mediators in Human Skin Fibroblasts Treated with Lipopolysaccharide. Molecules.

[22] Zaragozá C., Villaescusa L., Monserrat J., Zaragozá F., Álvarez-Mon M. (2020). Potential Therapeutic Anti-Inflammatory and Immunomodulatory Effects of Dihydroflavones, Flavones, and Flavonols. Molecules.

[23] Neil J.E., Brown M.B., Williams A.C. (2020). Human skin explant model for the investigation of topical therapeutics. Sci. Rep..

[24] Boisnic S., Branchet M.C., Gouhier-Kodas C., Verriere F., Jabbour V. (2018). Anti-inflammatory and antiradical effects of a 2% diosmin cream in a human skin organ culture as model. J. Cosmet. Dermatol..

[25] Moraes Silva M.A., Nelson A., Bell-Syer S.E., de Jesus-Silva S.G., Miranda F. (2024). Compression for preventing recurrence of venous ulcers. Cochrane Database Syst. Rev..

